# Preventive chemotherapy for the control of strongyloidiasis in school-age children: Estimating the ivermectin need

**DOI:** 10.1371/journal.pntd.0009314

**Published:** 2021-04-15

**Authors:** Donal Bisanzio, Antonio Montresor, Michael French, Richard Reithinger, Paola Rodari, Zeno Bisoffi, Dora Buonfrate

**Affiliations:** 1 RTI International, Washington DC, United States of America; 2 Epidemiology and Public Health Division, School of Medicine, University of Nottingham, Nottingham, United Kingdom; 3 Department of Control of Neglected Tropical Diseases, World Health Organization, Geneva, Switzerland; 4 Department of Infectious–Tropical Diseases and Microbiology, IRCCS Sacro Cuore Don Calabria Hospital, Negrar, Verona, Italy; 5 Department of Diagnostics and Public Health, University of Verona, Verona, Italy; Emory University, UNITED STATES

## Abstract

**Background:**

*Strongyloides stercoralis* is a soil-transmitted helminth (STH) that affects approximately 600 million people worldwide. Interventions targeting *S*. *stercoralis* have not been implemented yet. Specific treatment (ivermectin) could be included in already ongoing preventive chemotherapy (PC) campaigns targeting other STHs. The aim of this study was to estimate the quantity of ivermectin needed for an integrated STH/*S*. *stercoralis* control program.

**Methododology/Principal findings:**

Our study estimates the number of school- age children (SAC) (the main focus of STH deworming campaigns) in need of PC with ivermectin. The normal approximation of the binomial distribution was adopted to calculate the hypothetical prevalence distribution in each endemic country. Considering prevalence thresholds for PC equal to 10%, 15%, and 20%, we estimated the number of SAC in need of treatment. We adjusted the estimates accounting for ivermectin distributed in lymphatic filariasis and onchocerciasis elimination programs and excluded from our calculation areas where *Loa loa* is endemic.

The global number of SAC that should be targeted in PC campaigns was estimated at 283.9 M (95% CI: 163.4–368.8), 207.2 M (95% CI: 160.9–380.7), and 160.7 M (95% CI: 86.6–225.7) when the threshold for intervention was set to 10%, 15%, and 20%, respectively. India, China, Indonesia, Bangladesh, and Nigeria accounted for about 50% of the global SAC would have to be covered by PC intervention.

**Conclusions/Significance:**

Our analysis may support endemic countries to evaluate the ivermectin quantity needed for integrating strongyloidiasis in the existing STH programs. These estimates might also show to generic drug manufacturers the size of the potential market for ivermectin and encourage its production.

## Author summary

Strongyloidiasis affects about 600 million people worldwide, causing substantial morbidity. Large-scale programming to control strongyloidiasis is currently not implemented. We estimate that 161–284 million school-age children should be enrolled in deworming campaigns to substantially reduce the prevalence of strongyloidiasis. Our estimates might help endemic countries in estimating the ivermectin supply needed for the implementation of control programs for strongyloidiasis. As the availability of the drug at an affordable cost is a main issue, we hope that our estimates would encourage the production of generic ivermectin, showing the extent of the potential market.

## Introduction

*Strongyloides stercoralis* is a soil-transmitted helminth (STH) that globally is distributed in disadvantaged areas, where absent or inefficient sanitation causes the contamination of the soil with feces of infected individuals [1]. According to recent estimates, around 613.9 (95% CI: 313.1–910.1) million people could be infected worldwide, with South East Asia, Africa, and Western Pacific being the regions with the highest prevalence [2].

The infection is acquired through direct penetration of the skin by the infective larvae, and can lead to substantial related morbidity, such as abdominal pain, urticarial, respiratory symtoms [3]. Moreover, immunosuppressed individuals can develop disseminated strongyloidiasis, which is caused by the spread of larvae all over the body and can lead to death [3].

While control programs for the other STHs (i.e. *Ascaris lumbricoides* [roundworm], *Trichuris trichiura* [whipworm], *Ancylostoma duodenale* and *Necator americanus* [hookworms]) based on preventive chemotherapy (PC) [4], the large scale distribution of anthelmintic to population at risk, are already ongoing in several countries [5], they are not currently implemented specifically for *S*. *stercoralis*. This is mostly due to a previous underestimation of the prevalence and clinical burden, caused by the diagnostic challenge posed by the infection of *S*. *stercoralis* [6]. Indeed, Kato-Katz and microscopy examination of stool samples have an extremely low sensitivity for the detection of this parasite, which should better be investigated using other stool-based techniques (such as Baermann method, agar plate culture, polymerase chain reaction) or serology [7].

In light of the new evidence about the burden of strongyloidiasis [2], the World Health Organization (WHO) has recently included the control of strongyloidiasis among the objectives of the 2021–2030 Neglected Tropical Diseases (NTDs) Roadmap [8]. Among the new targets, the WHO recommends the implementation of control programs for *S*. *stercoralis* in school-age children (SAC), entailing the addition of ivermectin (IVM) to the distribution of benzimidazoles [9].

Recent work demonstrated that the geographical distribution of *S*. *stercoralis* and hookworm not only overlaps in many countries, but also follows a defined correlation [10]. This is caused by the biological characteristics of the two nematodes, which share the same route of transmission, and proliferate in areas where inadequate sanitation results in the contamination of the soil with human feces [6]. Hence, the integration of *S*. *stercoralis* control activities in PC programs already in place for hookworm seems a reasonable approach. Further, this option would benefit from taking advantage of the existing programmatic infrastructures, staff, and logistics. However, albendazole and mebendazole, the two drugs alternatively deployed presently in STH PC, have limited efficacy against *S*. *stercoralis* [11]. Single dose IVM showed a cure rate of 86% (95% CI 79–91) in a recent randomized controlled trial [12], with multiple doses not showing a higher efficacy. Hence, the inclusion of *S*. *stercoralis* in the PC programs for STHs would entail the addition of a single dose of IVM to the current strategy. At present, IVM is donated only in the context of the mass drug administration (MDA) programs for the elimination of lymphatic filariasis (LF) and onchocerciasis (OV). Indeed, one of the main impediments to the implementation of PC control programs for *S*. *stercoralis* is the limited availability of IVM at affordable cost.

The aim of the study was to estimate the quantity of IVM needed for an integrated STH / *S*. *stercoralis* control program, in order to provide a basis for negotiation with producers of generic ivermectin for a possible donation or preferencial price for large scale programs. The method applied considered three different all-age possible prevalence thresholds of infection and that PC programming entails treatment of the populations at risk without individual diagnosis, so many uninfected individuals are expected to be treated.

## Methods

### Target population

The target population of this study was SAC (5–15 years of age) living in 97 countries listed as in need for STH PC by the WHO [13]. We selected SAC because many endemic countries already have deworming programs providing drug treatment for children through PC MDA campaigns. Thus, logistically, as an initial step for the control of strongyloidiasis, it would be comparatively inexpensive for a country to include IVM treatment in school deworming programs where albendazole or mebendazole are already distributed. Use of IVM is not recommended in children under 5 years of age [14].

### Data dictionary

#### Strongyloidiasis prevalence

We use national-level prevalence figures of strongyloidiasis as estimated in a previous study for the 97 countries included in the study [2]. The database provides infection prevalence for each country estimated for the year 2017. Given that no endemic country has performed large-scale programming targeting strongyloidiasis, the 2017 prevalence estimates should not have changed significantly during the last three years.

#### Demographic data

Demographic data of each country was obtained from World Bank Open Data website [15]. The downladed data included SAC population and the fraction of population living in rural areas for the year 2019. PC MDA with IVM has been performed in some of the selected countries in the context of MDAs for LF or OV. Thus, populations covered by a PC targeting LF and/or OV likely do not need an additional intervention targeting strongyloidiasis. Hence, we obtained from the WHO the number of individuals included in PC programs using IVM between 2000 and 2018.

#### Estimate number of PC target population

We arbitrarily chose three different strongyloidiasis prevalence points as possible thresholds that would mandate PC MDA (10%, 15%, and 20%) to calculate the number of SAC to be covered with PC. Note, we did not have data of strongyloidiasis prevalences at subnational level to calculate the fraction of the population with prevalence above the thresholds. To estimate the binomial distribution of country prevalence, we used the normal approximation of the binomial distribution [16]. When a binomial distribution is symmetric, its density distribution can be approximated by a normal distribution N(μ, σ) with μ = np and σ = $\sqrt{npq}$, where n is the number of trials, p is the prevalence and q = 1-p. Thus, we made the assumption that the prevalence of strongyloidiasis follows a symmetric binomial distribution. This means that in a population with a mean strongyloidiasis prevalence of 10%, half of the SAC would belong to a fraction of the population with prevalence above this threshold and would need to be covered by PC. Similarly, in a country with a mean strongyloidiasis prevalence below 10%, less than half of the population would need PC. Using the obtained binomial distribution from the approximation, we calculated the fraction of population living in areas with prevalence above the three selected PC thresholds. Using countries’ age distribution, we estimated the number of SAC. We also assumed that if the prevalence were zero, nobody would meet the threshold for PC. Thus, we created three known estimates of the percentage of the population needing to be covered by PC. Using a linear model, we estimated a polynomial function to calculate the fraction of the population above the PC thresholds, given the mean strongyloidiasis prevalence.

### Adjusting for LF / OV MDA and presence of Loa loa

The estimate of SAC to treat with PC was adjusted by taking in account the fraction of children who may be covered by LF / OV MDA (and therefore already receiving IVM) and those who live in areas with *Loa loa* circulation (who should not receive IVM, based on the risk of developing severe encephalopathy [17]). While the OV PC data provide the number of SAC treated, the LF PC data do not provide the number of treatments broken down by age group. To estimate the number of SAC already covered by LF MDA, we partitioned the number of treated people by applying the countries’ age-group distribution; this assumes that LF MDA equally covered all age groups in any given country. We merged the number of SAC treated during LF / OV PC MDAs to estimate the number of children already treated with IVM. The estimates of SAC requiring treatment with IVM taking in account *Loa loa* presence was performed by using the estimates adjusted by LF / OV MDA and removing all countries in which *Loa loa* is endemic. The list of *Loa loa* endemic countries was based on the map published in Zoure et al. (2011) [18]. As we were not able to extrapolate the map’s high resolution data, we decided to remove from our estimates all children living in *Loa loa* endemic countries.

## Results

**Fig 1:**
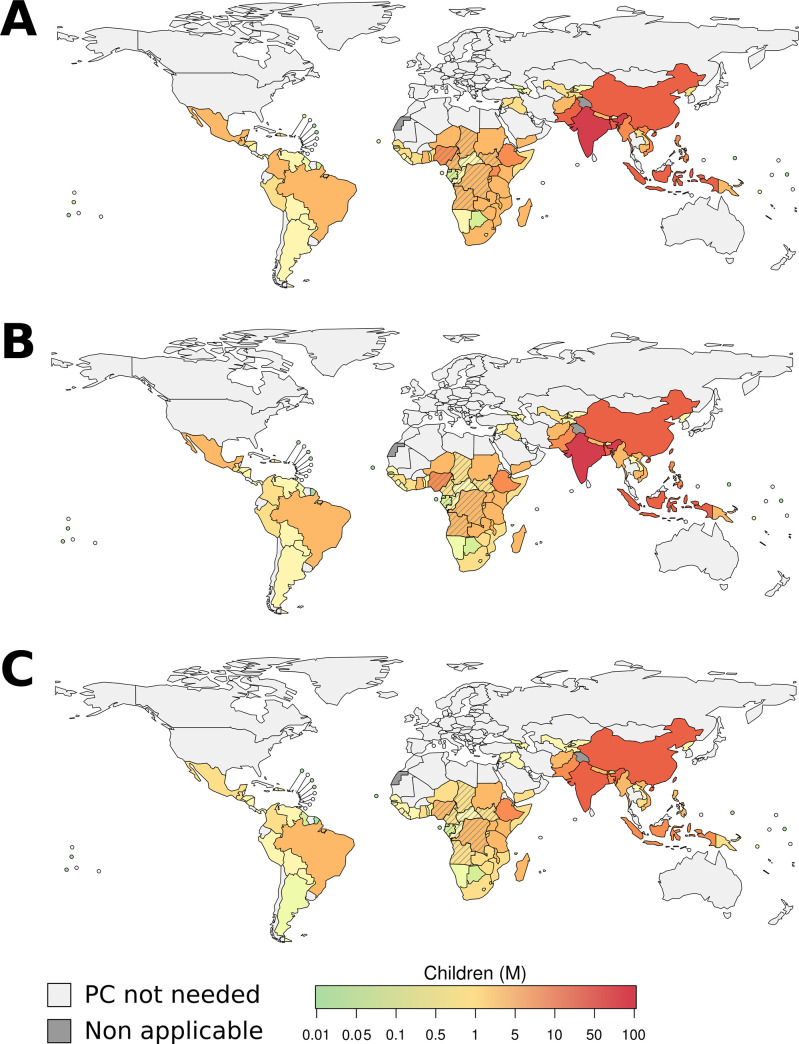
Number of SAC to be enrolled in PC intervention at country level, when the intervention threshold was set to 10%, 15%, and 20% strongyloidiasis prevalence. The map only shows estimates for countries for which WHO has suggested PC for other STHs. The PC coverage was calculated taking into account LF and ONCHO coverage. Barred countries are *Loa loa* endemic. The base layer of the map was retrieved from 2020 World Health Organization.

Based on our estimates, the global number of SAC that should be targetted in PC MDA campaigns (using 2018 population data) is 283.9 M (95% CI: 163.4–368.8), 207.2 M (95% CI: 160.9–380.7), and 160.7 M (95% CI: 86.6–225.7) when the intervention strongyloidiasis prevalence threshold was set to 10%, 15%, and 20%, respectively. Thus, by increasing the intervention threshold from 10% to 20% the number of SAC who should be covered by PC MDA decreased by 43.4% (95% CI: 38.8–47.7%). The region with the highest number of estimated SAC to cover with PC was South-East Asia (SEAR), with 128.9 M (95% CI: 75.3–164.0), 94.9 M (95% CI: 74.4–171.1), and 74.1 M (95% CI: 40.2–103.3) SAC when intervention threshold was set to 10%, 15%, and 20% prevalence, respectively (Table 1). The African region (AFR) had the second highest number of SAC to cover with PC, which was equal to 81.2 M (95% CI: 46.5–106.2), 59.1M (95% CI: 45.7–109.3), and 45.7 M (95% CI: 24.6–64.4) (Table 1 and Fig 1).

**Table 1:** Global and regional number of SAC who live in areas with strongyloidiasis prevalence above PC intervention thresholds. The table contains estimates made by taking into account LF/OV intervention and *Loa loa* endemicity.

WHO Regions	STG-PC School-age population [million (9*5%* CI*)]	STG-PC School-age population [million (95% CI)] not covered by LF/OV-PC	STG-PC School-age population [million (95% CI)] not covered by LF/OV-PC and living in *Loa loa* non-endemic countries
*Threshold*: *10% prevalence*			
AFR	81.2 (46.5–106.2)	59 (33.7–77.3)	42.8 (24.4–56.3)
AMR	10.8 (6.3–13.9)	10.8 (6.3–13.9)	10.8 (6.3–13.9)
EMR	20.4 (11.2–28.2)	20.3 (11.1–28)	20.3 (11.1–28)
EUR	1.9 (1–2.7)	1.9 (1–2.7)	1.9 (1–2.7)
SEAR	128.9 (75.3–164)	128.9 (75.3–164)	128.9 (75.3–164)
WPR	40.7 (23.1–53.8)	40.7 (23.1–53.8)	40.7 (23.1–53.8)
WORLD	283.9 (163.4–368.8)	261.6 (150.5–339.7)	245.4 (141.2–318.7)
*Threshold*: *15% prevalence*			
AFR	59.1 (45.7–109.3)	42.8 (33.1–79.5)	31.1 (24.1–57.8)
AMR	7.9 (6.2–14.4)	7.9 (6.2–14.4)	7.9 (6.2–14.4)
EMR	14.5 (10.9–28.2)	14.4 (10.9–28.1)	14.4 (10.9–28.1)
EUR	1.3 (1–2.6)	1.3 (1–2.6)	1.3 (1–2.6)
SEAR	94.9 (74.4–171.1)	94.9 (74.4–171.1)	94.9 (74.4–171.1)
WPR	29.5 (22.7–55.1)	29.5 (22.7–55.1)	29.5 (22.7–55.1)
WORLD	207.2 (160.9–380.7)	190.8 (148.3–350.8)	179 (139.2–329.1)
*Threshold*: *20% prevalence*			
AFR	45.7 (24.6–64.4)	33.1 (17.8–46.7)	24 (12.9–33.8)
AMR	6.2 (3.3–8.6)	6.2 (3.3–8.6)	6.2 (3.3–8.6)
EMR	11 (5.8–15.8)	10.9 (5.8–15.7)	10.9 (5.8–15.7)
EUR	1 (0.5–1.5)	1 (0.5–1.5)	1 (0.5–1.5)
SEAR	74.1 (40.2–103.3)	74.1 (40.2–103.3)	74.1 (40.2–103.3)
WPR	22.7 (12.2–32.1)	22.7 (12.2–32.1)	22.7 (12.2–32.1)
WORLD	160.7 (86.6–225.7)	148 (79.8–207.9)	138.9 (74.9–195)

STG: *Strongyloides;* PC: preventive chemotherapy; 95% CI: 95% confidence interval; AFR: African Region; AMR: American Region; EMR: Eastern Mediterranean Region; SEAR: South-East Asian Region; WPR: Western Pacific Region; EUR: European Region

The lowest estimated number of SAC to cover with PC was in the European Region (EUR), where less than 2 M children should be enrolled in a PC MDA campaign given the three-intervention thresholds (Table 1 and Fig 1).

At the country level, India, China, Indonesia, Bangladesh, and Nigeria were shown to account for ~50% of the global SAC to be covered by PC MDA with IVM at selected intervention thresholds (Fig 1, and S1– S3 Tables).

The fraction of SAC that received IVM during LF / OV MDAs between 2013 and 2018 accounted for less than 10% of the total SAC to be covered by PC targeting strongyloidiasis (Table 1). Because more than 98% of the population receiving IVM through LF / OV MDAs resided in AFR, the estimates for this region fell by ~20% (Table 1), when we accounted for LF / OV PC coverage. Thus, the resulting estimates for AFR fell to 59.1 M (95% CI: 33.7–77.3), 42.8 M (95% CI: 33.1–79.5), and 33.1 M (95% CI: 17.8–46.7) for the three intervention thresholds (Table 1).

Given the possible side-effects of IVM treatment administrated to individuals infected by *Loa loa*, we also made estimates excluding all countries where *Loa loa* is endemic. Accounting for the LF / OV interventions and excluding *Loa loa* endemic countries, reduces the fraction of SAC to cover with PC in AFR by ~50%. This reduction resulted in an estimated number of SAC to cover with PC equal to 42.8 M (95% CI: 25.1–58.0), 31.1 M (95% CI: 24.1–57.8), and 24.1 M (95% CI: 12.9–33.8) when the PC threshold was set to 10%, 15%, and 20%, respectively (Table 1).

## Discussion

In 2018, the WHO PC MDA campaigns for STHs reached around 456 M SAC (and 120 M pre-SAC) [5]. According to our estimates, the global number of SAC who should receive PC with IVM for strongyloidiasis is smaller, and differs widely (from 161 to 284 M) in relation to different thresholds that would mandate a PC MDA to control strongyloidiasis. In Africa, the co-existence of control programs for LF / OV already distributing IVM is another factor that reduces the global need. The evidence that IVM supply needed is “relatively small”, might ease the acceptance of an integration of current STH PC with IVM.

Moreover, the combination albendazole and IVM entails additional benefits. Indeed, it demonstrated increased efficacy compared to PC with albendazole or mebendazole alone for the other STH [19]. This seems to be important in particular for *T*. *trichiura*, which showed an insufficient response in some areas [20]. The enhanced activity on the other STH, combined with the effect on *S*. *stercoralis*, presumbably results in further reductions in morbidity in SAC. In a community-based study [21], anemia was found to be significantly associated with both hookworm and *S*. *stercoralis* infection, and was significantly reduced in SAC after PC with albendazole and ivermectin. Nutritional issues and stunting have been also associated with *S*. *stercoralis* infection in children[21–23]. Finally, the integration of strongyloidiasis control with STH control programs also seems reasonable in the context of a WASH (water, sanitation, and hygiene) approach, which is recommended to improve sanitation and, thus, help achieve control of STH transmission [24].

Indeed, evidence on the long-term impact of PC with IVM on the prevalence of strongyloidiasis is mostly based on studies carried out in the context of MDAs for other nematodes, which concomitantly resulted in a reduction of the prevalence of *S*. *stercoralis* infection [25]. An indirect benefit was also recently observed in the context of community treatment of scabies in the Solomon Islands [26]. Cure rates over 90% were reported in SAC who received PC with IVM in Cambodia [27] and Ethiopia [28] in the context of specific pilot programs targeting *S*. *stercoralis*. However, both studies considered only a short timeframe (from 2 to 3 weeks) after the intervention.

From a clinical point of view, it would be advisable to implement strongyloidiasis control programs even when prevalence is at lower levels, as infected individuals remain at risk of dissemination and fatal outcome all life-long. Moreover, inclusion of the entire community in PC MDAs for strongyloidiasis might be more beneficial, as the prevalence of infection is higher in adults [2,29]. In principle, PC for strongyloidiasis should aim at the elimination of infection rather than at a reduction of the clinical burden, which is the target of the other STH [30]. However, this would require major efforts likely beyond the scope of currently available resources, and school-based programs represent a good opportunity to begin control activities for *S*. *stercoralis* now. As such programs mature and resources allowing, they then should aim to be scaled up to include the whole community in a next step.

A limitation of our analysis is that our estimation was done at country level. The inclusion of data at district level might refine the figures, in particular when considering *Loa loa* endemic areas, as here we excluded the whole endemic countries from a possible PC with ivermectin, but this might not be needed for specific districts within an endemic country. However, these estimates do not aim to be the only basis on which countries should decide to implement control programs for *S*. *stercoralis*, and it is indeed recommended to conduct surveys in specific areas to evaluate possible interventions. These estimates aim to be the basis for an appraisal of cost constraints and benefits for public health purpose at country level. Indeed, *Strongyloides* endemic countries may take the decision to implement the PC according to availability of funds, and the threshold for PC implementation could be revised through the years, so that the number of SAC included could gradually increase.

Clearly, the implementation of a PC program for strongyloidiasis in STH programming does not merely entail the addition of IVM. Other operational processes and costs should be considered, such as additional training of staff / teachers who would administer the drug (the dose of which is weight-dependent). However, the integration within the STH programs would reduce the costs for logistics, that would be partly covered.

Of paramount importance for the beginning of strongyloidiasis PC MDAs would be the availability of IVM. If an extension of the current donation of IVM outside LF / OV elimination programs is not possible, at least it would be recommended to secure availability of generic IVM at affordable costs for strongyloidiasis PC. Some progress is going on in this regard, with producers of generic IVM applying for WHO pre-qualification and leaving room for possible reduction of costs for drug supply for programs against strongyloidiasis. Other drugs might also be considered as alternatives to IVM in the future; moxidectin in particular might be non-inferior than IVM against *S*. *stercoralis* [31]. It should also be considered that in many endemic countries individual diagnosis and treatment are seldom feasible; hence, in practice, access to IVM would only be possible through a PC program. Our estimates of the size of the IVM need to control strongyloidiasis should incentivize potential IVM manufacturers to produce the drug and facilitate the access of IVM at affordable cost—a crucial first step toward the implementation of large-scale programming to control strongyloidiasis.

## Supporting information

S1 Table SAC to be covered by PC MDA with IVM at country level, for a prevalence threshold of 10%. (XLSX)

S2 Table SAC to be covered by PC MDA with IVM at country level, for a prevalence threshold of 15%. (XLSX)

S3 Table SAC to be covered by PC MDA with IVM at country level, for a prevalence threshold of 20%. (XLSX)
